# Editorial: Proglucagon-Derived Peptides

**DOI:** 10.3389/fendo.2021.776871

**Published:** 2021-11-11

**Authors:** Peter R. Flatt, Filip K. Knop, Andrei I. Tarasov

**Affiliations:** ^1^ School Biomedical Sciences, Ulster University, Coleraine, Northern Ireland, United Kingdom; ^2^ Center for Clinical Metabolic Research, Gentofte Hospital, University of Copenhagen, Hellerup, Denmark; ^3^ Steno Diabetes Center Copenhagen, Gentofte, Denmark; ^4^ Department of Clinical Medicine, Faculty of Health and Medical Sciences, University of Copenhagen, Copenhagen, Denmark; ^5^ Novo Nordisk Foundation Center for Basic Metabolic Research Faculty of Health and Medical Sciences, University of Copenhagen, Copenhagen, Denmark

**Keywords:** proglucagon-derived peptides, L-cell, alpha-cell, obesity, diabetes, intestinal function, metabolism, therapeutics

## Proglucagon-Derived Peptide Research Topic

**Figure 1:**
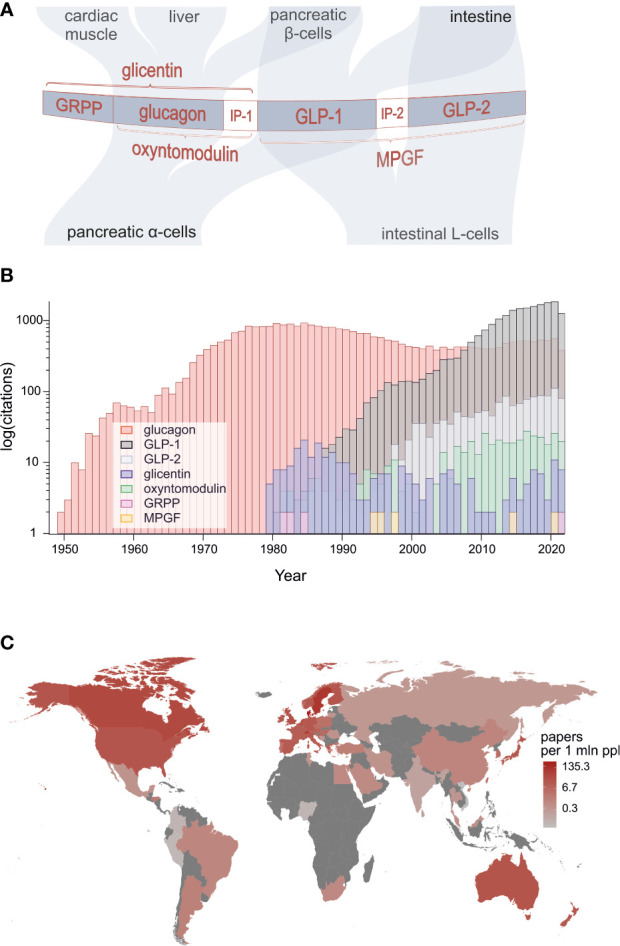
**(A)** Structure of proglucagon, post-translational processing and the major targets of constituent bioactive peptides: glucagon, GLP-1, GLP-2, oxyntomodulin, glicentin and glicentin-related pancreatic polypeptide. **(B)** Numbers of peer-reviewed publications on proglucagon-derived peptides showing particularly strong surge of research on GLP-1. **(C)** Publication count by geographical location divided by population of each country (source: PubMed).

Identification of the proglucagon gene at the beginning of the 1980s marked a huge breakthrough in research that would lead to discovery of a family of gene products that play a multitude of roles in regulation of feeding, metabolism and gastrointestinal function [**Figure 1A** (1–4)]. The peptide family members are also emerging players in pathophysiology and therapy of obesity and diabetes as well as several related metabolic disorders plus short bowel syndrome.

Surprisingly, it was found that glucagon-like immunoreactivity and subsequently the proglucagon protein encoded by the glucagon gene are present not only in the alpha-cells of the pancreatic islets but also in enteroendocrine L-cells of the intestine (5). Thus as shown in **Figure 1A**, in the pancreas, the precursor is processed by prohormone convertase 2 (PC2) to generate glucagon and glicentin-related pancreatic polypeptide (GRPP), whereas in L-cells, prohormone convertase 1/3 (PC1/3) processing results in production of glicentin, oxyntomodulin, glucagon-like peptide 1 (GLP-1) and glucagon-like peptide 2 (GLP-2). However, recent research challenges this tissue selectivity, demonstrating that under certain circumstances, alpha-cells appear to produce GLP-1, oxyntomodulin and glicentin whereas the intestinal L-cells may be a source of glucagon (4, 6).

Less controversial but more remarkably, GLP-1 was found to exert a plethora of physiological actions on intestine, pancreatic islets, brain and other tissues which are exploited in therapeutic approaches to obesity, diabetes, cardiovascular disease and neurodegenerative disorders using stable synthetic analogues or inhibitors of GLP-1 degradation (4). Indeed, GLP-1 mimetics and inhibitors of dipeptidyl peptidase 4 (DPP-4), an enzyme that renders GLP-1 and other proglucagon family members inactive by cleaving off N-terminal amino acids (7), are now well-established therapeutic agents. GLP-2 has also been found to be metabolically active and plays key role in stimulating intestinal growth. This has been exploited by development of N-terminally stabilised GLP-2 analogues for treatment of short bowel syndrome (3). GLP-2 has also been shown recently to inhibit gall bladder emptying in man, thereby promoting replenishment of bile stores following feeding (8).

During the past decade, it has been discovered that far from being inert, oxyntomodulin acts as a dual activator of GLP-1 and glucagon receptors with potential for promoting weight loss and glycaemic control (9). Evidence is emerging that another proglucagon-derived peptide, glicentin, may also have hitherto poorly appreciated physiological roles and utility as biomarker for intestinal or metabolic diseases (10).

As evident from above, we are in a fascinating and highly active era of research on proglucagon-derived peptides that is attracting considerable academic and industry interest geared towards increasing knowledge and the fight against the epidemic of obesity, type 2 diabetes and related disorders.

Under this Research Topic, we have assembled original research articles and reviews on many aspects of the biology, function, pathophysiology and therapeutic potential of post-translational products of the proglucagon gene, including:

➢*Glucagon*
➢*Glucagon-like peptide 1 *
➢*Glucagon-like peptide 2 *
➢*Oxyntomodulin*
➢*Glicentin*
➢*Glicentin-related pancreatic polypeptide (GRPP)*

In total, we have gathered 24 contributions from 117 leading scientists working in 13 different countries across the globe. The extent of this broad participation is testimony to the rising world-wide interest in research on proglucagon-derived peptides which is evidenced by the number of annual publications returned over time gathered using PubMed when searching for outputs using specific peptides as keywords (**Figures 1B, C**). A particularly strong upsurge in research on GLP-1 is evident with annual outputs on this peptide exceeding those published on glucagon since 2008. Interest in GLP-2 is also rising and recent evidence suggests that it has positive actions on bone.

A short appreciation of the papers included in our special collection on proglucagon-derived peptides is given below.

## History and Structural Aspects of Proglucagon-Derived Peptides

**Figure 2:**
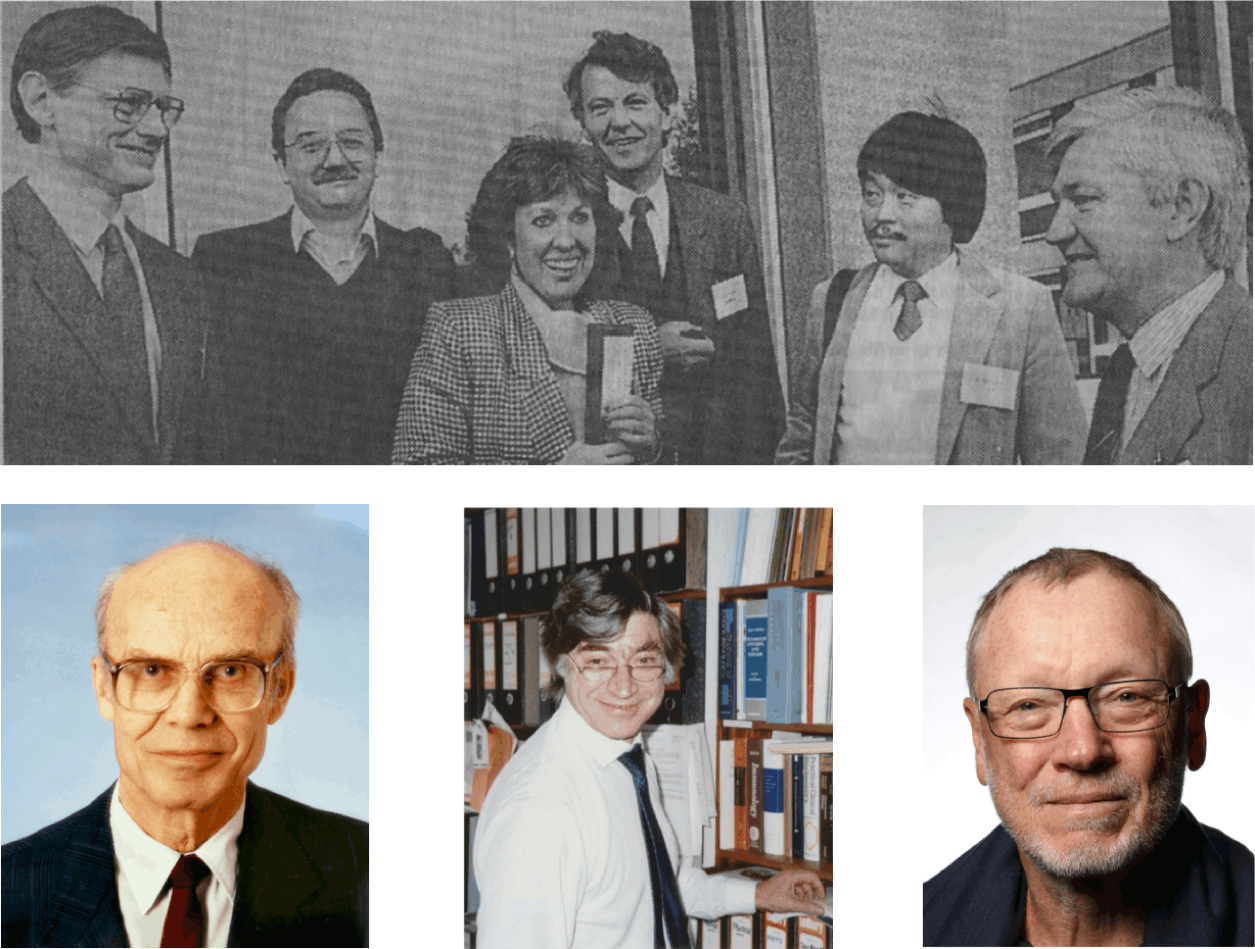
Contributors to some of the early milestones on proglucagon-derived peptides. Top panel (from left): Professor Steve Bloom, Professor J. Michael Conlon, Professor Julia Polak, Professor Flemming Stadil, Professor Kazuhiko Tatemoto and Professor Keith Buchanan pictured at the Annual Meeting of Bayliss & Starling Society, held in Belfast 1987 (reproduced with permission from Belfast Telegraph). Bottom panels (from left): Professor Bo Hellman (1996), Professor Vincent Marks (1989) and Professor Jens Holst (2020). BH by Lennart Nilsson, VM by PRF and JJH by Ricky Molloy. All photographs reproduced with permission.

The collection of papers starts with an historical perspective of studies on the N-terminal domain of proglucagon by Michael Conlon who together with Steve Bloom, Keith Buchanan, Jens Holst, Vincent Marks, Ellis Samols and others, including Roger Unger and Isobel Valverde, pioneered much of the early work in the 1970s on the proglucagon derived peptides (reviewed by Conlon and Marks in 5,11,12). Members of this family were picked up by antibodies raised against glucagon and with glucagon-like immunoreactivity. Some of the major milestones during this period are summarised in **Table 1** and several of the early pioneers in the field are shown in **Figures 1**, **2**. Thanks to the subsequent advent of molecular biology techniques and the additional pioneering efforts of Joel Habener, Daniel Drucker and others in the 1980s (1, 4, 41), the constituent bioactive peptides glucagon, GLP-1 and GLP-2 are now well recognised yet, as pointed out by Michael Conlon, the possible functional roles of oxyntomodulin, glicentin and GRPP are only just becoming elucidated.

**Table 1:** Some of the major milestones in the discovery, secretion and physiology of proglucagon-derived peptides with focus on pre-molecular biology era.

Year	Author(s)	Milestone	Reference(s)
1907	Lane	Distinguished alpha- and beta-islet cells	(11)
1923	Collip	Commented on initial hyperglycaemic effect of pancreatic extracts	(12)
1923	Kimball/Murlin	Discovered and named glucagon	(13)
1948	Sutherland/de Duve	Identified enteroglucagon	(14)
1956	Bromer	Structural elucidation of glucagon	(15)
1959	Unger	Glucagon radioimmunoassay	(16)
1962	Hellman/Hellerström/Unger/Madison	Islet alpha-cells recognised as site glucagon synthesis	(17, 18)
1962	Marks/Samols	Insulin-releasing action of glucagon	(19, 20)
1964	McIntyre/Holsworth/Turner	Demonstrated enhanced insulin release with oral glucose – the incretin effect	(21)
1965	Marks/Samols	Feeding increases circulating GLI	(22)
1967	Samols/Marks	Circulating GLI persists in humans following pancreatectomy	(23)
1970s	Valverde/Holst/Buchanan/Conlon	Heterogeneity of gut GLI, measurement of circulating GLI	(24–28)
1970s	Unger/Gerich Grodsky	Elucidation of glucagon physiology	(29, 30)
1972	Bloom	Greater villus growth in enteroglucagonoma (presumably action of GLP-2)	(31)
1973	Tager/Steiner	Characterization of oxyntomodulin, namely proglucagon (33–69)	(32)
1976	Unger	Importance of glucagon in diabetes	(33)
1982	Lund/Habener/Bell	Molecular biology elucidates proglucagon gene: glucagon, GLP-1, GLP-2, glicentin oxyntomodulin, GRRP	(34–36)
1987	Holst/Mojsov/Weir	Cleavage of GLP-1 (1–37) to GLP-1 (7–36) and demonstration of its potent insulin releasing activity	(37, 38)
1987	Bloom	Physiological insulinotropic action GLP-1 in man	(39)
1993	Mentlein	Degradation of glucagon family peptides, including GLP-1 (7–36) *in vitro* by DPP-4	(40)
1990s	Holst/Nauck/ Drucker	Elucidation of GLP-1 physiology	(41–44)
1994	Steiner	Role of PC2 in proglucagon processing to glucagon in islet alpha-cells	(45)
1995	Deacon/Holst	GLP-1 (9–36) - major metabolite in man, opening way for use of DPP-4 inhibitors and stable forms of GLP-1 for diabetes therapy	(46)
1996	Brubaker	Role of PC1/3 in differential proglucagon processing gut	(47)
1996	Drucker	Trophic action of GLP-2 in gut – opening way for future therapeutic use of GLP-2 analogues in short bowel syndrome	(48)

We acknowledge that many investigators have contributed to advances in research on proglucagon-derived peptides and we apologise for any obvious omissions. Interested readers are referred to several excellent reviews for detailed consideration of early work and the advances made after identification of the proglucagon gene: These include articles by Conlon, Holst, Drucker, Muller, Marks and their co-authors: 1,2,3,4,5,11,12,13. DPP-4, dipeptidyl peptidase 4; GLI, glucagon-like immunoreactivity; GLP-1, glucagon-like peptide 1; GLP-2, glucagon-like peptide 2; GRPP, glicentin-related pancreatic polypeptide; PC1/3, prohormone convertase 1/3; PC2, prohormone convertase 2.

In the following papers dealing with structural aspects, David Irwin has exploited advances in gene technology to document variations in the evolution and sequences of proglucagon and receptors for its post-translational peptide products. Such comparative data may provide important information regarding unforeseen functional aspects of these peptides. Indeed, the work of Lindquist et al. considers the mutational landscape of proglucagon-derived peptides, highlighting how small structural changes may contribute to the pathophysiology of glucose intolerance and the efficacy of GLP-1-based therapies.

## Alpha-Cell Function and Secretion of Proglucagon-Derived Peptides

Turning to the alpha-cell, Dhanvantari and Asadi consider signalling pathways involved in the regulation of glucagon secretion with focus on direct effects of glucose plus increasingly recognised intra-islet autocrine and paracrine mechanisms. This involves not only classical islet hormones, insulin and somatostatin, but also glucagon and other products of proglucagon processing such as GLP-1, oxyntomodulin and GRPP. The article by Wei He et al. specifically addresses the significance of islet GLP-2 production and its effects on islet inflammation. Islets also contain substantial amounts of peptide YY (PYY) known to interact with neuropeptide Y (NPY) receptors. Lafferty et al. demonstrate important actions of NPY1 receptor activation on islet structure with positive effects on transdifferentiation of alpha-cells to beta-cell phenotype.

## L-Cell Function and Secretion of Proglucagon-Derived Peptides

Although also expressing the proglucagon gene, the L-cell differs substantially in its biology to the alpha-cell. The paper by Kuhre et al. is a provocative and timely discourse on ‘What Is an L-Cell and How Should Its Secretory Mechanisms Be Studied?’ Thus, it is increasingly clear that enteroendocrine cells are promiscuous and may express several structurally distinct regulatory peptides as well as biologically active post-translational degradation products. In the case of the L-cell, this includes PYY, GLP-1, GLP-2, glicentin, oxyntomodulin and, perhaps under certain circumstances, glucagon and other PC1/3-generated products. Furthermore, as discussed by Kuhre et al., the hormone composition of L-cell differs markedly depending on anatomical location and the L-cell type is not a homogeneous population. They suggest that L-cells are sub-classified depending on their differential peptide contents as well as their differential expression of nutrient sensors, which ultimately determine the secretory responses to different stimuli. The most frequently used experimental models for functional L-cell studies are discussed also, with the conclusion that a comprehensive understanding can only be built on results from a combination of models.

In rat studies, Hira et al. showed that GLP-1 was released immediately following feeding from the distal intestine and that dietary protein played a critical role in determining postprandial GLP-1 response in rats. Using mice, Hunt et al. demonstrate the importance of dietary fibre in the maintenance of intestinal weight, colonic L-cell secretion and intestinal integrity. In humans, Jonsson et al. reveal a limited role of endogenous bile acids in the acute regulation of GLP-1 secretion after Roux-en-Y gastric bypass surgery. This draws attention to the importance of other mechanisms in the marked and therapeutically beneficial increase of circulating GLP-1 that is observed consistently following such procedures. With an eye on the possibility of therapeutically exploiting endogenous GLP-1 stores, Kuhre et al. used mouse and rat models to explore the mechanisms through which melanocortin-4 receptor agonists stimulate GLP-1 secretion and conclude that the effect was indirectly mediated or possibly restricted to colonic L-cells. Further studies exploring the metabolic benefits of GLP-1 secretagogues in type 2 diabetes, possibly in combination with oral DPP-4 inhibitors, are clearly warranted.

## Islet Effects of Proglucagon-Derived Peptides

The next series of papers deals with the actions of proglucagon-derived peptides. Starting with the beta-cell, Marzook et al. consider substantial progress in our understanding of GLP-1 receptor signalling and trafficking, such as the perpetuation and termination of signalling within endosomal compartments. It emerges that the reprogramming of GLP-1 receptor endocytosis and post-endocytic sorting represents a useful means, using biased GLP-1 receptor agonists, to achieve distinct signalling patterns at different subcellular locations with important therapeutic implications. Ahrén et al. considers studies on insulin secretion and glycaemic control in mice with knock-out of GLP-1 receptors. The relatively mild phenotype observed draws attention to arousal of important compensatory mechanisms. Whether these include hyperactivity of L-cells and effects of raised concentrations of other proglucagon-derived peptides or interactions beyond the GLP-1 receptors are interesting possibilities.

## Extrapancreatic Effects of Proglucagon-Derived Peptides

Proglucagon-derived peptides exert numerous extrapancreatic effects which are fundamental in their physiology. For example, GLP-1 targets multiple cell types mediating diverse effects on many body systems. Puddu and Maggi document the emerging role of caveolin-1 in the action of GLP-1. They suggest that the interaction between GLP-1 receptor and Cav-1 is necessary not only for receptor trafficking to the cell membrane, but also for activation of different components of the intracellular signalling pathway. This is interesting given that augmentation of GLP-1 action can be envisaged as therapeutically useful. Similarly, Lee et al. showed that inhibition of G protein-coupled receptor kinases using small molecule inhibitors increased the insulinotropic action of GLP-1 and potentiated DPP-4-mediated suppression of circulating glucose in mice. The benefit of activation of GLP-1 pathways is well established in the treatment of obesity and type 2 diabetes. The paper by Li et al. considers additional uses for Alzheimer’s disease, hypertension and non-alcoholic steatohepatitis with important mediation by neuroprotective and anti-inflammatory actions. GLP-2 is used for treatment of short bowel syndrome, but its intestinotrophic effect has been suggested to promote colonic neoplasia. Hunt et al. report that glucagon receptor knock-out mice that exhibit inappropriately raised circulating concentrations of GLP-1 and GLP-2 are not more susceptible to azoxymethane/dextran sodium sulphate-induced tumours. Using enzyme resistant [Gly^2^]GLP-2, Mieczkowska et al. also noted enhanced collagen post-processing and crosslinking maturation in murine osteoblasts, indicating possible therapeutic benefit in osteoporosis or bone fragility generally. Interestingly, [D-Ala^2^]GLP-1 or glucagon were without effect.

## Dietary Measures Utilising Proglucagon-Derived Peptides to Improve Metabolic Control

It is evident from above, that proglucagon-derived peptides have found significant therapeutic applicability. Kamruzzaman et al. consider gut-based strategies to reduce postprandial glycaemia in type diabetes, focussing on stimulation of GLP-1 release. Further to this, Smith et al. describe a successful randomised control trial in obese and lean men of postprandial glucose responses following ingestion of a novel, ready-to-drink shot containing low dose of whey protein. Such dietary adjuncts are being explored in many studies currently based on the ability of various protein digests to trigger the release of GLP-1 and other metabolically beneficial gut peptides such as PYY and cholecystokinin (CCK).

## Proglucagon-Derived Peptides as Therapeutics

Although dietary measures may be beneficial in mild cases of type 2 diabetes, most patients likely to benefit therapeutically from activation of proglucagon-derived pathways will require drug intervention. Lafferty et al. address the current status of proglucagon-derived peptides as therapeutics. This includes glucagon, GLP1, GLP-2, oxyntomodulin, glicentin, GRRP as well as unimolecular multi-agonist peptides which activate receptors for GLP-1, glucagon and GIP. The therapeutic application extends to diabetes, obesity, cardiovascular and neurodegenerative diseases, short bowel syndrome, osteoporosis, polycystic ovary syndrome and hypoglycaemia. Hope et al. further consider the strong potential of GLP-1/glucagon receptor co-agonism as a treatment strategy for obesity. They discuss the importance of relative balance of co-agonism, the positive effect of glucagon on energy balance and how its natural hyperglycaemic actions are countered by the insulinotropic action of GLP-1. Tanday et al. demonstrate the value of upregulated unimolecular GLP-1/CCK receptor signalling in rodent obesity-diabetes, indicating the therapeutic potential offered by recapitulation of the interlinked pathways naturally activated by feeding. Exploitation of such an approach requires imagination of peptide chemists to come up with viable peptide analogues. In this vein, it is notable that He et al. describe a simple method for conjugation of two proglucagon peptide analogues via added cysteine residues.

## Concluding Remarks

As evident from the above, research on proglucagon-derived peptides has delivered significant outcomes and had real measurable societal impact. Much has been discovered since elucidation of glucagon, the exploitation of antibody-based technologies by radioimmunoassay and immunocytochemistry and elucidation of the proglucagon gene. The more we dig into the biology of this influential peptide family, the more questions we turn up that need to be answered. We extend our thanks to the authors for their timely contributions, to the reviewers for their efforts in evaluating the manuscripts and to you the readers who we hope will gain knowledge and inspiration from this timely collection of papers.

## Author Contributions

All authors have contributed to this editorial and approved it for publication.

## Conflict of Interest

The authors declare that the research was conducted in the absence of any commercial or financial relationships that could be construed as a potential conflict of interest.

## Publisher’s Note

All claims expressed in this article are solely those of the authors and do not necessarily represent those of their affiliated organizations, or those of the publisher, the editors and the reviewers. Any product that may be evaluated in this article, or claim that may be made by its manufacturer, is not guaranteed or endorsed by the publisher.
